# Impact of cuts to local government spending on Sure Start children’s centres on childhood obesity in England: a longitudinal ecological study

**DOI:** 10.1136/jech-2020-216064

**Published:** 2021-06-21

**Authors:** Kate E Mason, Alexandros Alexiou, Davara Lee Bennett, Carolyn Summerbell, Ben Barr, David Taylor-Robinson

**Affiliations:** 1 Public Health, Policy and Systems, University of Liverpool, Liverpool, UK; 2 Department of Sport and Exercise Sciences, School of Medicine and Health, Durham University, Stockton-on-Tees, UK

**Keywords:** child health, obesity, social epidemiology, public health policy

## Abstract

**Background:**

Childhood obesity is rising in disadvantaged areas in England. Sure Start children’s centres provide community-based services for children <5 years and their parents, including many services that can support healthy weight, directly or indirectly. Since 2010, austerity-driven cuts to local authority (LA) budgets have led to substantially reduced public expenditure on Sure Start services. We assessed whether childhood obesity prevalence has increased more since 2010 in those areas in England that experienced greater cuts to spending on Sure Start.

**Methods:**

This longitudinal ecological study covers the period 2010/2011–2017/2018. Our exposure was LA expenditure on Sure Start, using Department for Education data. Our main outcome was LA obesity prevalence at age 4–5 years, using National Child Measurement Programme data. We used fixed-effects panel regression to quantify the association between change in spending and change in the prevalence of childhood obesity.

**Results:**

Spending on Sure Start children’s centres decreased on average 53% over the study period, with deeper cuts in more deprived LAs. Each 10% spending cut was associated with a 0.34% relative increase in obesity prevalence the following year (95% CI 0.15% to 0.53%). We estimated there were an additional 4575 children with obesity (95% CI 1751 to 7399) and 9174 overweight or obese (95% CI 2689 to 15 660) compared with expected numbers had funding levels been maintained.

**Conclusions:**

Cuts to spending on Sure Start children’s centres were associated with increased childhood obesity. With deprived areas experiencing bigger spending cuts, reinvesting in these services may, alongside wider benefits for child development, contribute to reducing inequalities in childhood obesity.

## Background

**Figure 1:**
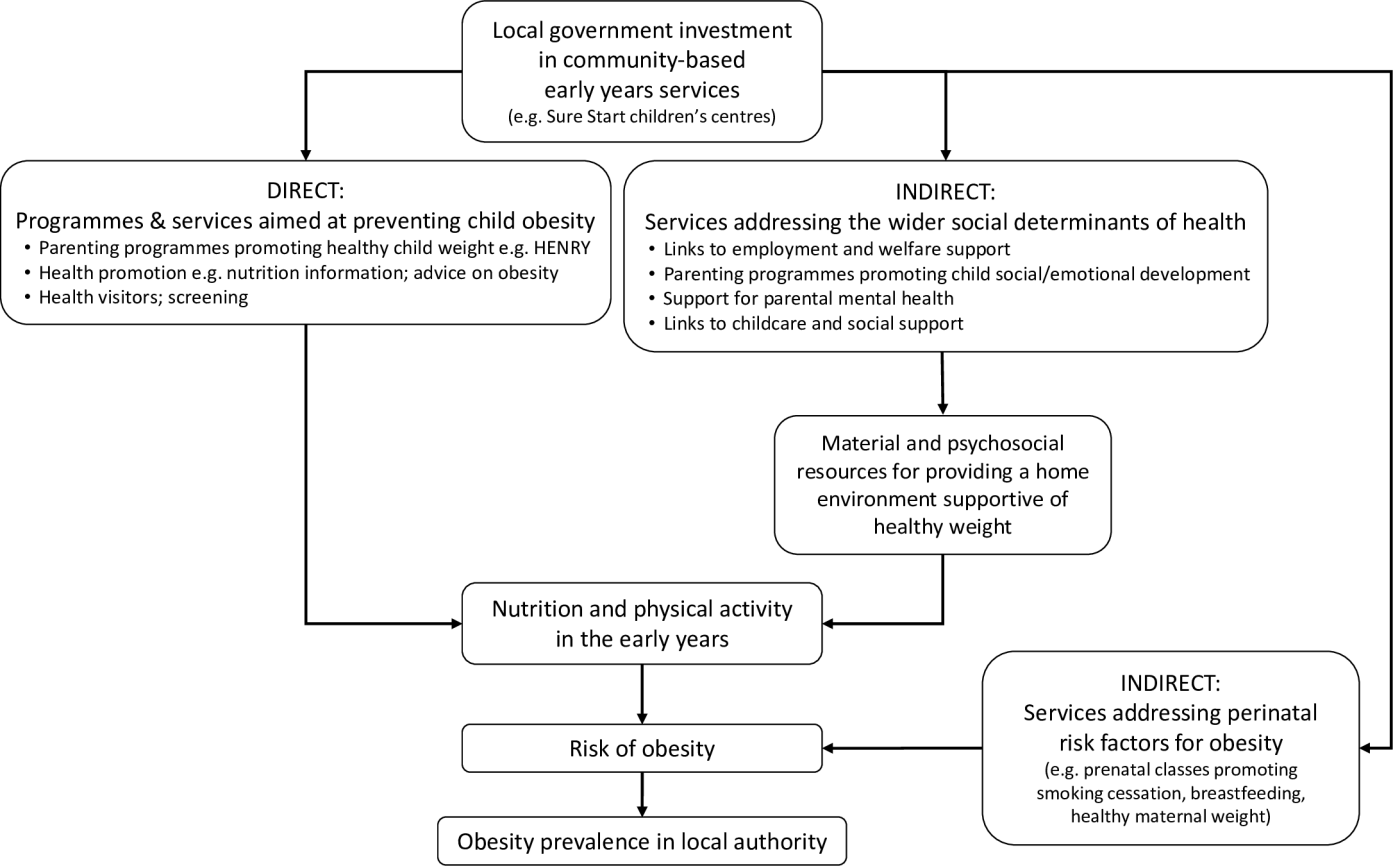
Logic model showing direct and indirect pathways from investment in community-based early year services such as Sure Start children’s centres to the local prevalence of childhood obesity.

In England, 1 in 10 children at reception and one in five children at the end of primary school are living with obesity. After recently stabilising, childhood obesity prevalence is rising again in disadvantaged areas of England.^1^ Obesity in childhood has consequences for adult health and life chances as well as child health.^2^ The causes of childhood obesity are multiple and complex, a fact belatedly recognised in the UK Government’s 2020 obesity strategy^3^ and no single intervention is likely to solve the issue.^4^ Some modifiable pathways to childhood obesity may operate through investment in community-based early years services, which can support healthy nutrition and physical activity in numerous direct and indirect ways (figure 1). In England, local authority-run Sure Start children’s centres have been an important source of community-based services targeting children aged under 5 and their families, including: parenting programmes; promotion of breastfeeding, good nutrition and active play; prenatal and health visitor services; early learning and links to childcare and links with employment and welfare support for parents, especially in more deprived communities. At the initiation of the Sure Start scheme in 1999, obesity prevention and the narrowing of health inequalities were explicit aims of the centres.^5^

Evaluation of the early phase of the Sure Start scheme—when centres were concentrated in the most deprived areas and tasked principally with reducing social inequalities in child outcomes—showed mixed results.^6^ Nevertheless, the evaluation did find that children at age 5 living in neighbourhoods with access to a Sure Start centre were less likely to be overweight than 5-year olds living in comparable areas without a centre.^7^ More recently, a report by the Institute for Fiscal Studies (IFS) examined the child health impact of increasing access to Sure Start children’s centres from the start of the programme to its peak coverage in 2009/2010.^8^ They found evidence of fewer hospitalisations among children with greater access to children’s centres, and the benefits were concentrated among children in more deprived areas. However, in exploratory analyses presented in the same report, no association with BMI or obesity was detected, although data limitations led the authors to interpret those results cautiously.

Austerity measures adopted by the UK government from 2010 resulted in large cuts to local authority (LA) budgets, forcing many councils to prioritise spending on statutory child protection services at the expense of nonstatutory services, including Sure Start children’s centres.^9 10^ Severely reduced investment has led not only to the closure of many children’s centres but also to a reduction in the range and quality of services offered by surviving centres.^11^ Cuts to spending on these services have recently been linked to worse child development outcomes,^12^ and other childhood outcomes such as overweight and obesity may also have been affected. These cuts may also have contributed to the recent widening of the obesity gap between least and most deprived children, and to the reversal of improving trends seen in some areas.

Previous studies evaluating the effects of Sure Start focused on the rollout and scale-up of the programme and were not designed to investigate the impact of cuts to spending on these services. In this study, we make use of the natural policy experiment of rapidly changing funding for early years services in England to assess whether: (i) changes in spending after 2010 were associated with changes in the prevalence of obesity at school reception (age 4–5 years), (ii) the association varied by deprivation and (iii) the association varied by the trend in obesity prevalence in the years preceding the study period.

## Methods

### Data sources and study design

In this longitudinal ecological study, we used publicly available data covering the period 2010/2011–2017/2018. We used LA data on obesity prevalence at reception (children aged 4–5 years) from the National Child Measurement Programme (NCMP).^13^ Height and weight of children in state-run mainstream primary schools are measured each year, and measurements are assigned to LAs based on children’s home postcode. Reliable LA-level data are available from Public Health England for each academic year from 2007/2008 onwards. We used gross LA expenditure data for children’s services from the section 251 LA outturn reporting published annually by the Department for Education (DfE), collated in the Place-based Longitudinal Data Resource.^14^ These reports include measures of LAs’ annual spending, disaggregated across a range of children’s services including Sure Start children’s centres. Inconsistencies over time in the way the data are reported limited the availability of comparable annual data prior to the 2010/2011 financial year. We compiled data for upper tier LAs—the level of local government at which children’s social care services are delivered. Of the 152 upper tier LAs in England, we used data from 147, excluding two LAs with very few children and three LAs with implausible expenditure values.

### Measures

#### Outcomes

Our primary outcome was the percentage of children in reception with obesity. The NCMP defines obesity as BMI (kg/m^2^) greater than or equal to the 95th centile of a UK reference sample of measurements gathered in 1990, taking into account age and sex. As a secondary outcome, we analysed overweight (including obesity) (BMI ≥ 85th centile of the growth reference). To allow for lagged effects on child body weight, we lagged the outcome by one year in our primary analysis, such that, for example, spending in the financial year April 2016–March 2017 is linked to obesity prevalence in the academic year September 2017–August 2018. As the NCMP measurement of children can take place at any time throughout the academic year, in effect this lag from the beginning of the preceding financial year may cover anywhere between 17 and 29 months for a given school in the NCMP.

#### Exposure

Our exposure variable was total expenditure per child in the DfE’s spending category ‘Sure Start Children’s Centres and Early Years’. The majority (roughly two-thirds) of this spend each year was on individual Sure Start centres, with smaller amounts devoted to area-wide services delivered through Sure Start centres, centre management costs, and, from 2013/2014 onwards, ‘other early years expenditure’. This ‘other’ subcategory typically accounted for ~18% of the annual total. Associated documentation describes the broader category as designed to capture total LA spending on Sure Start, so we retained the ‘other’ subcategory in our annual totals. Herein, Sure Start spending refers to the entire category of spending. Expenditure values were population-normalised by dividing them by the number of under-5 children in that LA in the same year, as estimated by the Office for National Statistics and scaled to 2018/2019 prices, using the Office for Budget Responsibility’s 2020 GDP deflator.^15^

#### Potential effect modifiers

To assess whether the association between spending cuts and obesity prevalence varied by the level of deprivation in a LA, we tested for an interaction between spending on Sure Start and the average 2015 Index of Multiple Deprivation score^16^ of each LA.

To assess whether the association between spending cuts and obesity prevalence varied according to the LA’s trend in obesity prevalence prior to austerity, we tested for an interaction between spending and the average annual change in obesity prevalence over the period from 2007/2008 to 2010/2011 inclusive. This was of interest because if Sure Start services had been part of a successful local obesity prevention strategy in some areas, then we might expect areas where child obesity prevalence had been declining to be more sensitive to spending cuts than areas where prevalence was stable or rising.

#### Potential confounders

Our analytical approach, described below, estimates the within-area change in obesity associated with changes in spending, using fixed-effects regression, which removes the need to account for time-invariant confounders, that is, factors that vary *between* LAs but not *within* them over the relatively short time scale of the study (eg, demographic characteristics). Of concern are only time-varying potential confounders, that is, LA-level factors that change over time and influence both the level of spending and the obesity risk of children. We identified a priori three such variables: local economic conditions, child poverty and spending on other children’s services. The relationships between variables are described in the online supplemental material and depicted in a directed acyclic graph in the online supplemental figure S1. We used gross disposable household income (GDHI) as an annual measure of local economic conditions^17^; data from Her Majesty's Revenue and Customs (HMRC) on the proportion of children living in low-income families in each year to capture child poverty; and the sum of all non-Sure Start expenditure in the DfE data set described above to capture spending on other children’s services.

10.1136/jech-2020-216064.supp1 Supplementary data

### Statistical analysis

First, we assessed descriptive trends in obesity prevalence and spending between 2010/2011 and 2017/2018, overall and by the quintile of average deprivation of LAs. We then assessed the correlation between change in obesity and change in spending at the LA level.

For the main analysis, we used Poisson panel regression, with fixed effects for LAs and individual years, to estimate the mean within-LA relationship between spend and obesity while accounting for secular trends across all LAs. We modelled obesity as the number of children classified as obese, with the population denominator (measured children) as an offset variable to account for different population sizes, thereby effectively modelling the prevalence. We log-transformed the exposure variable to account for expected diminishing returns on investment. Model results are, therefore, interpretable as change in obesity prevalence relative to the percentage change in spend. To reflect the scale and direction of actual changes in spending over the study period, we report the results as the change in obesity prevalence associated with every 10% *decrease* in spending. We estimated robust SEs due to the clustering of data points over time within areas. To minimise confounding, we adjusted for GDHI (continuous); proportion of children in low-income households (continuous); and total spending on all other children’s social care services (log-transformed, continuous). From the model, we also estimated the excess number of children with obesity over the study period, by predicting the obesity prevalence if all LAs were funded at the 2010 mean for the entire study period.

We then separately estimated models with interaction terms between spend and continuous measures of each of the potential effect modifiers: deprivation and prior trend in obesity prevalence. We used a Wald test to assess the strength of evidence for effect modification by each. We estimated marginal effects of spend for quintiles of deprivation and representative values of pre-2010 obesity trend, using the ‘margins’ command in Stata V.14.2. Three additional LAs were excluded from the analysis of effect modification by pre-2010 obesity trend due to missing obesity data for earlier years. In all analyses, we limited the time series for spending and potential confounders to the period from 2010/2011 to 2016/2017 because data for GDHI and children in low-income families were not available for later years. As the outcome was lagged by 1 year, we used obesity data up to 2017/2018.

#### Negative control analysis

To assess whether any association detected in our primary analysis was due to unmeasured confounding, we conducted a negative control analysis using spending on services for older children as a control exposure. In a negative exposure control analysis, the main analysis is repeated substituting an alternative exposure variable that would not be expected to influence the outcome, but which has a similar confounding structure with respect to the outcome.^18 19^ Expenditure on ‘Young People’s Services’ is a separate category of spending from the same data set and pays for social care services targeting young people aged 13–19 years. If no association is observed between the negative control and obesity, a causal interpretation of the primary association is more plausible. If a non-null association is observed, it is likely that residual confounding is responsible for any observed association in the primary analysis.

### Robustness checks and secondary analyses

We performed several tests of the robustness of our findings to alternative model specifications (details in the online supplemental material). We also conducted secondary analyses for overweight and obesity combined, rather than obesity only.

## Results

### Descriptive statistics

**Figure 2:**
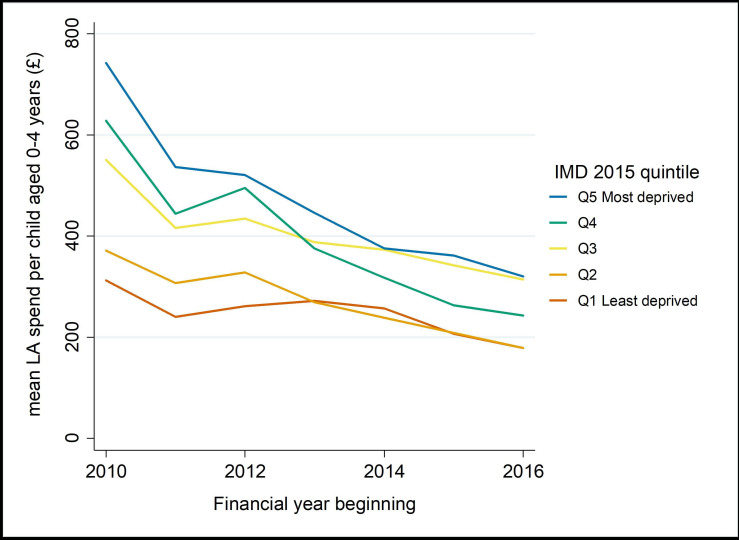
Mean local authority expenditure on Sure Start and early year services per child aged 0–4 years, by deprivation quintile, 2010/2011–2016/2017 (2018/2019 prices). IMD, Index of Multiple Deprivation; LA, local authority.

**Figure 3:**
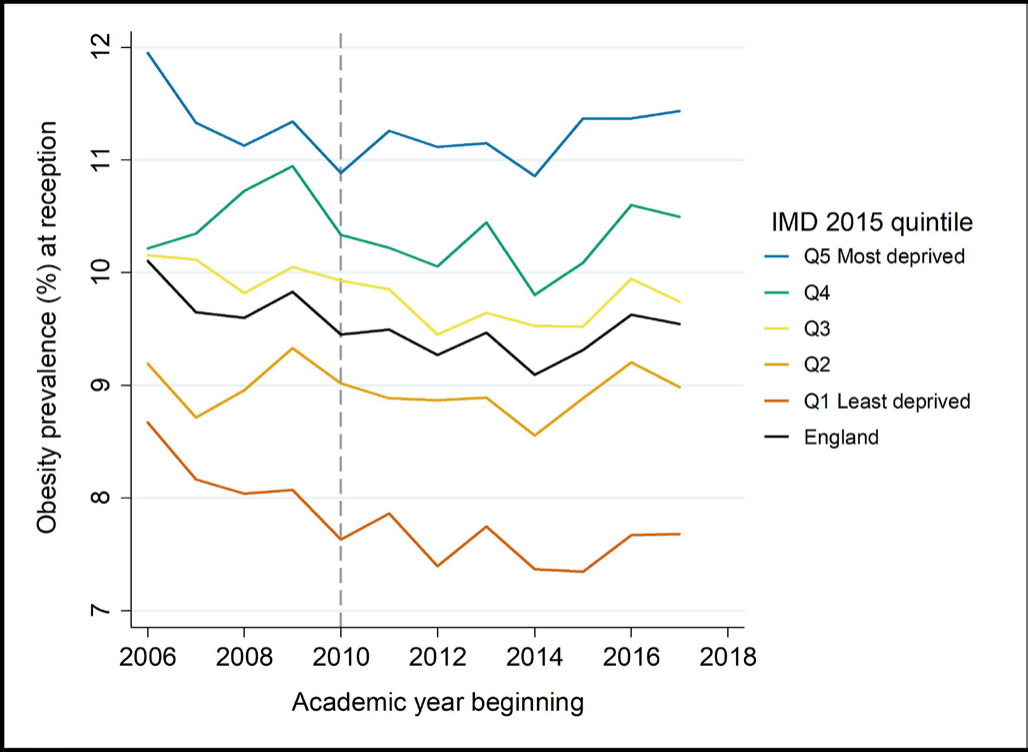
Trend in obesity prevalence (%) at school reception (age 4–5 years) by deprivation, England, 2006/2007–2017/2018. IMD, Index of Multiple Deprivation.

**Figure 4:**
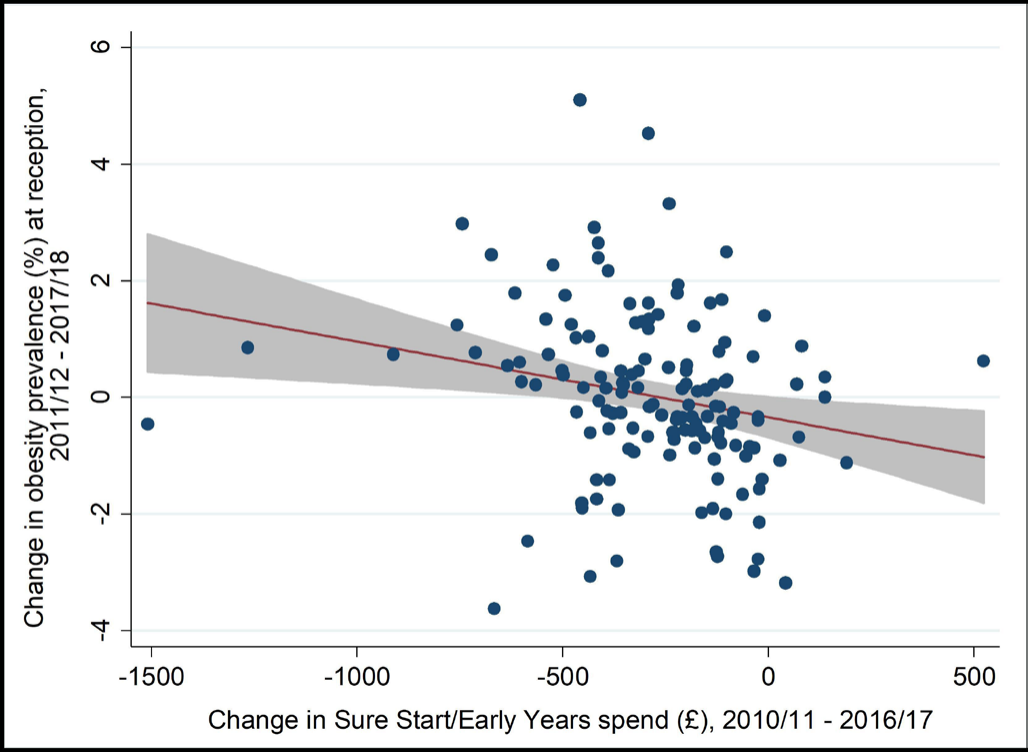
Correlation between change in spend and change in obesity prevalence.

Between 2010/2011 and 2016/2017, mean LA spending per child (0–4 years) on Sure Start and early years’ services decreased by 53% in real terms (figure 2; online supplemental table S1). Spending in the most deprived quintile of LAs decreased by £422 per child between 2010 and 2016, compared with £133 per child in the least deprived quintile. Annual real-term cuts to spending averaged 8.3% per year, varying from 6% in the least deprived quintile of LAs, to 11% in the most deprived quintile. Following a period of apparent decline from 2007/2008, obesity prevalence at reception has plateaued and more recently started to increase in some areas, particularly more deprived LAs (figure 3). Change in spend and change in obesity were weakly negatively correlated (r=−0.22) (figure 4).

### Primary analysis

#### Poisson analysis of the association between spending and obesity prevalence

**Figure 5:**
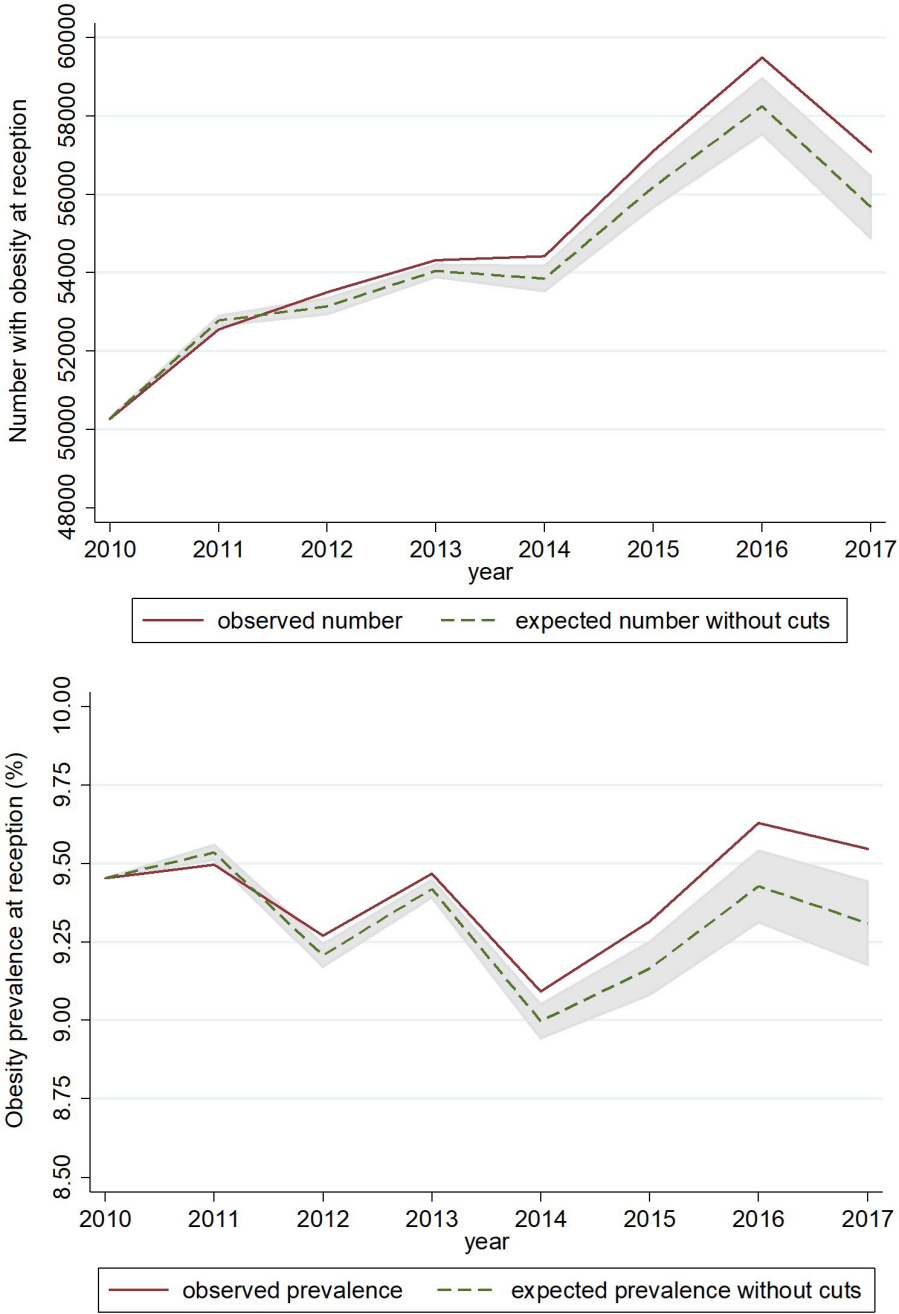
Observed number/prevalence of children with obesity vs number/prevalence expected in the absence of spending cuts.

On average, obesity prevalence increased more in areas with larger cuts to Sure Start spending. We estimated that each 10% spending cut in a financial year was associated with a 0.34% relative increase in obesity prevalence the following academic year (95% CI 0.15% to 0.53%, table 1). Over the study period as a whole, our model suggests an additional 4575 children with obesity (95% CI 1751 to 7399) compared with the expected number had funding levels remained constant at the mean 2010 level (figure 5). This corresponds to approximately 108 per 100 000 additional children per year with obesity.

**Table 1:** Association between local authority spending and obesity prevalence at reception (Poisson model)

	Relative change in obesity prevalence (95% CI)	P value
Sure Start and early years spending (10% decrease in spend, 2018/2019 prices)	0.34% (0.15% to 0.53%)	0.001
Spend (10% decrease) x prior obesity trend		0.002
Average annual change in prevalence 2007/2008–2010/2011*		
0.5% decrease	0.57% (0.34% to 0.80%)	
No change	0.29% (0.10% to 0.48%)	
0.5% increase	0.01% (−0.29% to 0.30%)	
Spend x deprivation		0.372
*Quintile of Index of Multiple Deprivation*		
Q1 (least deprived)	0.49% (0.26% to 0.73%)	
Q2	0.22% (-0.08% to 0.52%)	
Q3	0.18% (−0.23% to 0.58%)	
Q4	0.36% (0.06% to 0.66%)	
Q5 (most deprived)	0.59% (0.05% to 1.13%)	
Youth services spending*—negative control* (10% decrease in spend, 2018/2019 prices)	−0.03% (−0.22 to 0.16)	0.761

All models adjusted for the proportion of children in low-income families, gross disposable household income and spending on all other child social care services. 95% CIs are based on robust standard errors.

*Table shows effect estimates for representative values of prior trend in obesity prevalence.

#### Effect modification by LA deprivation level

The association did not vary by deprivation level of the LA (P_interaction_=0.372; table 1) when assessed on the multiplicative scale using the log-linear Poisson model. Using a linear fixed effects model in sensitivity analyses, effect modification by deprivation was observed on the difference scale, but there was no clear trend across the deprivation gradient (online supplemental table S2).

#### Effect modification by prior trend in childhood obesity prevalence

The association between spending and obesity within LAs was stronger in areas where obesity prevalence had been falling up to 2010/2011 (P_interaction_=0.002; table 1). The average annual change in obesity prevalence for the period 2007/2008–2010/2011 ranged from a 2.2 percentage point decrease to a 0.9 point increase. We estimated that in areas where the average annual change in obesity prevalence to 2010/2011 was a decrease of half a percentage point, a 10% spending cut after 2010 was associated with a 0.57% relative increase in obesity prevalence (95% CI 0.34% to 0.80%; table 1); that is, reversing the downward trend seen prior to the spending cuts. In contrast, in areas where obesity prevalence up to 2010/2011 had been increasing by half a percentage point each year on average, a 10% spending cut in subsequent years was not associated with any change in obesity prevalence (0.01%, 95% CI −0.29% to 0.30%; table 1).

### Negative control analysis

As expected, there was no evidence of an association between spending on services for older children and obesity prevalence in the school reception year. A 10% cut in spending on youth services was associated with a 0.03% relative decrease in obesity prevalence at reception (95% CI −0.22% to 0.16%). We can, therefore, have greater confidence that the estimate for the effect of spending on Sure Start is not residually confounded.

### Robustness checks

Results were very similar for overweight and obesity combined (online supplemental table S4 and figure S2). Results were sensitive to the lag time between spending and obesity—the magnitude of the main association was smaller for both no lag and a 2-year lag, and for the 2-year lag the 95% CI was much wider and no longer excluded zero (online supplemental material). Using a linear rather than Poisson regression model, we estimated a main effect equivalent in magnitude (online supplemental table S2). Excluding any of the potential confounders had only a small impact on our main point estimates (online supplemental table S3).

## Discussion

This study provides evidence that recent cuts to local government spending on Sure Start children’s centres were associated with an increasing prevalence of obesity in 4–5-year olds. Since 2010, for every 10% cut in expenditure in a given financial year, we estimate that LA obesity prevalence among children starting school increased by 0.34% the next academic year. This amounts to an additional 4575 children with obesity, as a result of spending cuts, over the study period. The relationship observed was more pronounced in areas where obesity prevalence had been falling prior to the spending cuts. Although the relationship was of a similar magnitude across all levels of area deprivation, the far greater cuts to spending in poorer areas mean a disproportionate absolute impact on obesity in these areas.

In this natural policy experiment, we evaluated the impact of rapidly changing early years spending across England using high-quality objectively measured NCMP outcome data, with high levels of coverage (>93% eligible pupils each year of the study period), providing robust estimates of obesity prevalence at LA level. Our analytical approach excludes the possibility of confounding by time-invariant differences between LAs but assumes no residual time-varying confounding. Changing levels of local investment in other efforts to reduce childhood obesity (not delivered through Sure Start centres) could confound the relationship. Since public health budgets were not under the remit of LAs until 2013, we lacked data to adjust for this spending. We also relied on HMRC data on children in low-income families, known to underestimate child poverty.^20^ Therefore, to assess the risk of residual confounding, we conducted a negative control analysis, investigating whether obesity prevalence at reception was implausibly associated with the level of spending on older children’s services. The null finding offers reassurance that the main results are not biased by residual confounding. The accuracy of expenditure data is uncertain and may vary within and between LAs. There are some unexplained discrepancies between the DfE data we used and a similar (but less granular) data set produced by the Ministry of Housing, Communities and Local Government. Reporting differences over time may be a source of information bias.

We have assessed this research question at the level of LAs, the level at which investment in these services occurs. Nevertheless, ecological analyses risk introducing bias by aggregating individual characteristics to the group level. Measuring obesity, deprivation and child poverty in the aggregate, at a relatively coarse geographical level, may obscure differential effects of spending cuts on particularly marginalised neighbourhoods and social groups. Our analysis is also constrained by the availability of time series of suitable covariate data. Depending on the mechanisms by which exposure to Sure Start most strongly influences obesity risk (eg, services affecting infants vs 2–4-year olds), we might expect a similar effect on obesity prevalence with a greater time lag. However, in our sensitivity analyses using a 2-year lagged outcome, the point estimate was attenuated. A wide CI around that estimate suggests that the analysis may have been underpowered to detect an effect for a longer lag. Expanding the data set when additional years of data become available would allow a more reliable exploration of alternative lag structures between variables.

A recent IFS study reported no association between access to Sure Start centres and childhood obesity.^8^ Our approach differs from that study in two important ways and is, thus, complementary. First, while the IFS report focused on the impact of Sure Start from its beginning to its (pre-austerity) peak in 2010, we have focused on the period after 2010, when investment in these services was in sharp decline. Second, we estimated the effect of *spending* on these services, while the IFS report looked at the effect of *geographical access* to children’s centres. Extreme budgetary pressures on local councils over the past decade have led not only to the closure of many children’s centres but also to reduced staffing and a curtailed range of services in surviving centres.^11^ While an important measure, geographical access to a children’s centre captures only the presence or absence of a centre in an area, not the volume or quality of surviving services. Our findings are somewhat consistent with the National Evaluation of Sure Start’s report on the impact on 5-year olds, which found lower risk of overweight (but not obesity) among children in areas with a Sure Start centre early in the rollout of the programme.^7^ A similar programme in the USA was also shown to lower obesity rates in teenage boys who had been exposed to the programme in their preschool years.^21^

Our findings suggest that cuts to LA spending on children’s centres since 2010 were associated with increases in childhood obesity, large enough to have seemingly reversed some gains made prior to the introduction of austerity measures. The scaling back of Sure Start, alongside cuts to public health budgets, has constrained the provision of child obesity prevention programmes that were delivered through some centres (eg, HENRY)^22^ and diminished the wider offer of services that can indirectly support healthy weight in childhood (among other benefits), as illustrated in figure 1. Our finding that effects of spending cuts were stronger in areas that had–before austerity–seen declining obesity prevalence, suggests disinvestment in Sure Start may be undermining strategic progress in the important goal of reducing the prevalence of childhood obesity.

The UK Government has declared its aim to ‘significantly reduce the gap in obesity between children from the most and least deprived areas by 2030’.^23^ Yet this gap continues to grow across all age and sex groups.^1^ Government plans to tackle the commercial determinants of obesity by restricting price promotions and advertising of unhealthy foods to children are important, overdue steps in the right direction. But there is no magic bullet; other efforts will be needed and must address poverty and deprivation as root causes of obesity.^24^ The recent update of the Marmot Review highlights Sure Start and children’s centres as exemplifying the proportionate universalism advocated in the original Marmot Review, whereby services are provided for entire communities but scaled and intensified for those with the greatest need.^25^ Our study suggests that reversing cuts to Sure Start—cuts that disproportionately affected deprived areas—could be an effective component in a wider, multifaceted approach to reducing the prevalence of childhood obesity and narrowing the existing deprivation gap.

Local governments face difficult decisions about the allocation of scarce resources. These necessarily involve weighing competing demands for investment to address multiple threats to children’s safety and well-being. Obesity is conservatively estimated to cost the NHS around £6 billion annually, not including wider societal costs such as lost productivity.^2^ Reinvestment in children’s centres has the potential to mitigate substantial downstream costs to society across the life course, not only from obesity prevented but also across many health and nonhealth domains. Across England, a return to 2010 levels of spending would involve reinvesting around £900 million per year above current levels. Alternative models for funding and service delivery may be able to achieve the same or better outcomes, for example, through involvement of the third sector, industry or philanthropy. Ongoing work commissioned by the Department for Education is reviewing research and practice evidence with the aim of developing tools to support decision-making by LAs in the strategic use of children’s centres.^26^ Our study contributes to that evidence base, demonstrating that broad early years support may help prevent obesity by the start of school and contribute to narrowing the obesity gap between the least and most deprived.

Further research with individual-level and small area data linked to LA spending may improve our insight into the impacts of spending cuts on childhood obesity and develop our understanding of the extent to which investment in services like Sure Start might measurably offset effects of poverty and other household factors on obesity in young children. Qualitative research involving centres and parents may shed light on which particular Sure Start services were driving our observed findings. Building on the earlier IFS study, similar analyses of health impacts of recent centre closures would be informative alongside our study.

In addition to other expected implications for young children and their parents, disinvestment in early years children’s services is likely to be undermining progress in tackling childhood obesity. Looking ahead to what is likely to be a period of increased financial pressure on local government due to the pandemic, if funding for these services cannot be restored, we may see obesity rates in young children increasing and inequalities in the early years and beyond widening further.

**What is already known on this subject**

- Since 2010, large cuts to English local authority budgets have forced many councils to reduce spending on Sure Start children’s centres, which deliver a wide range of services to support parents and young children. It is possible that these cuts have influenced childhood overweight and obesity, contributing to the observed widening of the obesity gap between least and most deprived children over recent years and reversing improving trends seen in some areas prior to 2010. Previous studies evaluating the effects of Sure Start on various child health and development outcomes were focused on the rollout and scale-up of the programme and were not designed to investigate the impact of cuts to spending on these services under the recent austerity programme.

**What this study adds**

- We made use of the natural policy experiment of rapidly changing funding for early years services in England and found that cuts to Sure Start spending after 2010 were associated with increased prevalence of obesity at school reception (age 4–5 years). We estimate that several thousand additional children became overweight or obese compared with the expected number if spending levels had remained at 2010 levels. These findings suggest that reinvesting in Sure Start children’s centres may be one way to address some of the root causes of childhood obesity and inequalities in obesity.

## Data Availability

Compiled data on local government expenditure used in this study are openly available to access at https://pldr.org/, or by contacting a.alexiou@liverpool.ac.uk. All other data are available in public, open access repositories, as cited.
