# Supplementary material for: Impact of cuts to local government spending on Sure Start children’s centres on childhood obesity in England: a longitudinal ecological study
Source: J Epidemiol Community Health. 2021 Jun 21;75(9):860–6. doi: 10.1136/jech-2020-216064 (PMC8372393; doi:10.1136/jech-2020-216064)
Supplement: Supplementary data [file jech-2020-216064supp001.pdf]

SUPPLEMENTARY MATERIAL

Impact of cuts to local government spending on Sure Start Children's Centres on childhood obesity in England: a longitudinal ecological study

KE Mason, A Alexiou, D Bennett, C Summerbell, B Barr, D Taylor-Robinson

Directed acyclic graph and description of potential confounders

Our use of fixed-effects regression to estimate the within-area change in obesity associated with changes in spending removes the need to account for time-invariant confounders. We identified three potential time-varying confounders and adjusted our models for these, reflecting the directed acyclic graph in Figure S1. First, local economic conditions will reflect need and also influence the level of revenue from business rates and central government, thus determining how much is available to be spent on children's services, while also influencing child health through the socioeconomic conditions of parents. We use Gross Disposable Household Income (GDHI) as an annual measure of this.[1] Second, the level of child poverty in an area is both an indicator of need for services and a determinant of child health. We use HMRC data on the proportion of children living in low-income families in each year to capture this. Third, spending on other services for children may also confound the association, especially since LA budgets are finite and constrained. For instance, more spending on statutory child protection services leaves less money available for discretionary services such as Sure Start, and spending on other services may influence child obesity directly or indirectly.

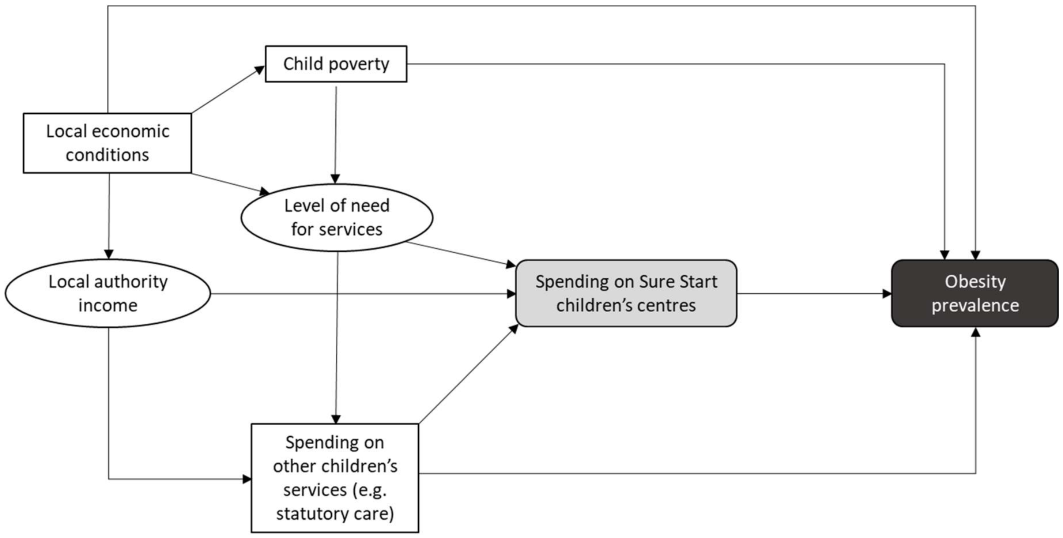

Figure S1. Directed acyclic graph of the relationship between spending on Sure Start and obesity at age 4-5 years.

Mason KE, *et al. J Epidemiol Community Health* 2021; 75:860–866. doi: 10.1136/jech-2020-216064

Table S1. Descriptive stats (N=147 Local Authorities)

	Mean (SD)	Absolute (and %) change from 2010
**Sure Start spend per child in 2010/11 (£ 2018/19 prices)**	524.23 (266.62)	
IMD Q1 (Least deprived)	312.25 (134.93)	
IMD Q2	371.09 (106.64)	
IMD Q3	550.90 (263.40)	
IMD Q4	627.70 (272.04)	
IMD Q5 (Most deprived)	742.17 (243.38)	
**Sure Start spend per child in 2016/17 (£ 2018/19 prices)**	247.92 (156.23)	-£276 (-53%)
IMD Q1 (Least deprived)	178.80 (89.28)	-£133 (-43%)
IMD Q2	178.18 (92.69)	-£193 (-52%)
IMD Q3	314.45 (219.82)	-£236 (-43%)
IMD Q4	242.76 (122.77)	-£385 (-61%)
IMD Q5 (Most deprived)	320.41 (158.35)	-£422 (-57%)
**Obesity prevalence in 2010/11 (%)**	9.61 (1.63)	
IMD Q1 (Least deprived)	7.60 (0.81)	
IMD Q2	9.15 (1.06)	
IMD Q3	9.98 (1.18)	
IMD Q4	10.38 (1.42)	
IMD Q5 (Most deprived)	10.81 (1.37)	
**Obesity prevalence in 2017/18 (%)**	9.65 (1.84)	+0.4%
IMD Q1 (Least deprived)	7.51 (1.20)	-1.3%
IMD Q2	8.97 (1.28)	-1.9%
IMD Q3	9.64 (1.16)	-3.5%
IMD Q4	10.53 (1.26)	+1.5%
IMD Q5 (Most deprived)	11.45 (1.44)	+5.9%
**Pre-2010 trend in obesity prevalence**	**n (%)**	
Areas with decrease or no change in % with obesity	92 (63%)	
Areas with increase in % with obesity	55 (37%)	

Robustness checks

Linear model rather than Poisson

To test the robustness of our findings to alternative model specifications we repeated the primary analyses using linear rather than Poisson regression models. When we used a linear model, a 10% decrease in spending was associated with an absolute change in obesity prevalence of 0.033 percentage points (95% CI: 0.015, 0.052; Table S1). In the context of the baseline (2010) prevalence this is very similar in magnitude to the Poisson model results (a relative increase of 0.34% from a base prevalence of 9.4% is 0.032 percentage points). In contrast to the Poisson analysis, with the linear model there was evidence of an interaction with deprivation that was not observed with the primary analysis (p=0.005). While the association appeared to be stronger in the most deprived areas, there was not a clear trend with increasing deprivation. This apparent contradiction is expected, because the absence of an interaction on the multiplicative scale (as for this log-linear Poisson model) implies the presence of an interaction on the difference scale (linear model) if both exposure and effect modifier are causally related to the outcome.[2] The multiplicative interaction detected with prior trend in obesity prevalence persisted on the difference scale.

Table S2. Association between local authority spending and obesity prevalence at reception (linear model)

	Absolute change in obesity prevalence, in percentage points (95% CI)	P value
Sure Start & Early years spending (10% decrease in spend, 2018/19 prices)	0.033 (0.015, 0.052)	<0.001
Spend (10% decrease) * pre-2010 obesity trend		0.002
Average annual change in prevalence 2007-2010[a]		
0.5% decrease	-0.053 (-0.077, -0.030)	
No change	-0.027 (-0.045, -0.010)	
0.5% increase	-0.002 (-0.026, 0.023)	
Spend*deprivation		0.005
IMD Q1 least deprived	-0.023 (-0.047, 0.000)	
IMD Q2	-0.007 (-0.030, 0.017)	
IMD Q3	-0.027 (-0.061, 0.008)	
IMD Q4	-0.049 (-0.073, -0.025)	
IMD Q5 most deprived	-0.082 (-0.136, -0.028)	
Youth services spending - *negative control* (10% decrease in spend, 2018/19 prices)	0.006 (-0.011, 0.022)	0.505

Alternative lags between exposure and outcome

We also examined the effect of not lagging the outcome, and of lagging the outcome by two years rather than one. In both cases we observed an attenuation of the association between spending and obesity, compared with our primary analysis with a one-year lag. For a 10% cut in spending, we estimated a 0.19% increase in obesity prevalence with no lag in the outcome (95%CI: 0.07%, 0.32%) and a 0.10% increase in obesity prevalence with a two-year lag (95%CI: -0.17%, 0.38%).

Alternative adjustment sets and alternative exposure definition

We estimated unadjusted and partially adjusted models to examine the influence of confounder adjustment on the main results. Excluding any of the potential confounders had only a small impact – all combinations from none to all covariates gave a point estimate somewhere between 0.33% and 0.41%, so our primary analysis errs on the conservative side. We also examined the effect of defining the exposure as total spend rather than per capita spend. The primary result was robust to operationalising spend without adjusting for the number of 0-4 year olds in the local authority. (Table S3)

Overweight at reception (including obesity) as outcome

In a set of secondary analyses we examined overweight and obesity as the outcome (defined as BMI greater than or equal to the 85th centile of the growth reference), rather than obesity only. Results were very largely unchanged when expanding the outcome definition to include overweight. Each 10% decrease in spend was associated with a 0.28% relative increase in prevalence of overweight or obesity (Table S4), and the model suggested an additional 9,174 children overweight or obese (95% CI: 2,689 – 15,660) over the study period compared with the expected number if spending had been held constant at the mean 2010 level (Figure S2). The negative control again showed the expected null association, and again the trend was stronger in areas where overweight and obesity had been declining prior to 2010, while no effect modification by deprivation was observed.

Mason KE, *et al. J Epidemiol Community Health* 2021; 75:860–866. doi: 10.1136/jech-2020-216064

Table S3. Associations between local authority spending on Sure Start and Early Years services and prevalence of obesity at reception, with alternative adjustment sets and alternative exposure definition

	Relative change in obesity prevalence (95% CI)
Unadjusted	0.40% (0.20%, 0.60%)
Adjusted for:	
GDHI	0.33% (0.15%, 0.52%)
% children in low income families	0.39% (0.17%, 0.61%)
Other children's services spending	0.41% (0.21%, 0.62%)
GDHI and % children in low income families	0.34% (0.14%, 0.53%)
GDHI & other children's services spending	0.34% (0.16%, 0.53%)
% children in low income families & other children's services spending	0.40% (0.18%, 0.61%)
Exposure=change in total spend rather than per capita spend	0.35% (0.16%, 0.54%)

Table S4. Association between local authority spending on Sure Start and Early Years services and prevalence of overweight (including obesity) at reception

	Relative change in overweight & obesity* prevalence (95% CI)	P value
Sure Start & Early years spending	0.28% (0.09%, 0.46%)	0.003
(10% decrease in spend, 2018/19 prices)		
Spend (10% decrease) * pre-2010 obesity trend		0.0314
Average annual change in prevalence 2007-2010		
0.5% decrease	0.38% (0.18%, 0.58%)	
No change	0.28% (0.09%, 0.46%)	
0.5% increase	0.17% (-0.04%, 0.39%)	
Spend*deprivation		0.6513
IMD Q1 least deprived	0.50% (0.27%, 0.72%)	
IMD Q2	0.14% (-0.11%, 0.38%)	
IMD Q3	0.15% (-0.16%, 0.46%)	
IMD Q4	0.26% (-0.04%, 0.55%)	
IMD Q5 most deprived	0.48% (0.03%, 0.92%)	
Youth services spending - *negative control*	-0.01% (-0.18%, 0.16%)	0.933
(10% decrease in spend, 2018/19 prices)		

*BMI greater than or equal to the 85th centile of the growth reference

Mason KE, *et al. J Epidemiol Community Health* 2021; 75:860–866. doi: 10.1136/jech-2020-216064

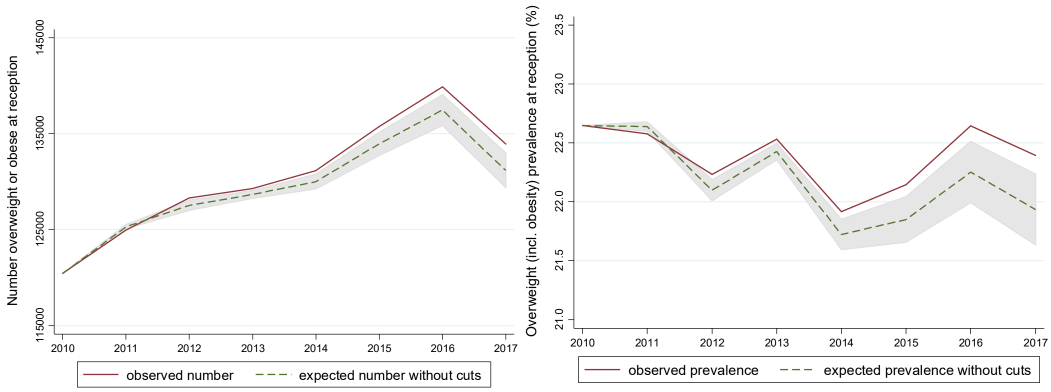

Figure S2. Observed number/prevalence of overweight (incl. obese) children vs number/prevalence expected in the absence of spending cuts

REFERENCES

1 Office for National Statistics. Regional gross disposable household income by local authority. https://www.ons.gov.uk/economy/regionalaccounts/grossdisposablehouseholdincome/datasets/regionalgrossdisposablehouseholdincomegdhibylocalauthorityintheuk (accessed 13 Oct 2020).

2 VanderWeele T. *Explanation in causal inference: methods for mediation and interaction*. Oxford University Press 2015.
